# Progress in Skin Diseases

**Published:** 1901-06-01

**Authors:** 


### Progress in Skin Diseases.

(*Continued from page 134.*)

**Bazin's Malady.**—Hutchinson [37] regards this affection as a scrofulous ulceration rather than as an erythema, but it is rarely seen in the presence of other tubercular diseases, pulmonary or glandular, with the exception of phlyctenular ophthalmia. As a rule the ulcers are confined to the legs, though occasionally others are seen on the scalp and on the back of the upper arm. Syphilitic forms indeed occur which can only be distinguished by the history in some cases. However, syphilitic cases are more often restricted to one leg, and show sores elsewhere; they are also more amenable to treatment.

**Tuberculides.**—Bœck [38] thinks the lesions are due to the absorption of toxin from tuberculous foci, as the bacillus can rarely be found in them and even the inoculation test often fails. Two groups may be distinguished, the superficial, such as L. scrofulosorum, and the deeper lesions, such as L. erythematosus and the nodular forms. Colcott Fox finds that histologically some diseases of both groups resemble tuberculous granulomata and show other evidence in the same direction. Darier, while acknowledging the difficulty of proof, considers the tuberculous origin is a fact, but would explain the contradictions by supposing that they are due to bacilli of feeble virulence killed by the tissues after setting up the lesions in question. Many authorities, however, deny that L. erythematosus is of tuberculous origin at all. Graham Little [39] recently showed three cases. In one there were papules on the buttocks, backs of arms, and face, which suppurated and slowly dried up, leaving dark stains. In two others similar papular pustules were widely scattered. A woman also presented two masses of granulation tissue of the size of half-a-crown on the arm, as well as some patches of scaly dermatitis showing faint white scarring. This he also regards as a form of tuberculous affection of the skin.

**Herpes Zoster.**—Traumatic herpes is discussed by Gaucher and H. Bernard.[40] Cases have been seen after a blow, a fall, or sprain, more often after an incised wound or amputation. The eruption may appear a few hours or some years after an injury; sometimes it occurs on the exact spot of the injury, sometimes over other branches of the same nerve. It has even followed dental operations. Winternitz,[41] in the treatment of zona, applies several folds of gauze soaked in absolute alcohol and covered by protective, which is bandaged over the affected area. He finds that it relieves the pain, causes the vesicles to heal without ulceration, and checks the rise of fresh eruptions. Du Castel [42] draws a graphic picture of the misery produced by recurrent herpes, mentioning a case where the eruption came out on the edge of the tongue every few weeks for five years. Like many such patients, the man was possessed by the idea of syphilis. The lesions, whether on the prepuce, the eyelids, the lips, or the mucus membranes are certainly not syphilitic, and should not be treated with mercury, which is definitely harmful. Simple herpes may occur once in a lifetime, after a fever or an injury, but in some persons it appears on the least malaise, while in a few it recurs regularly, quite irrespective of the general health. There is a form which appears with each menstruation in some women on various parts of the body, and another which recurs on the genital organs of the male, causing sometimes severe neuralgic pains. The latter type must be carefully distinguished from chancre and chancroids when seen in a patient for the first time, but it is apt to recur every few weeks, especially after fatigue or sexual excitement, to the great annoyance of the sufferer. The buccal form, which especially affects the edge of the tongue, is often seen in persons who have been cured of syphilis many years before, and is neither improved by syphilitic remedies nor followed by other syphilitic symptoms. The vesicles may be small and quickly destroyed by the surrounding moisture, so that minute ulcers, with sloughing shreds of epithelium, may be all that can be seen. The treatment of these forms of herpes is, unfortunately, most ineffective. Arsenic in large doses and the water of sulphur springs have relieved some patients, while care of the general health and the removal of all sources of irritation are recognised as important. The disease has one good feature—it never becomes the starting-point of a cancer.

Head[43] considers that herpes of the tongue is usually of the H. labialis type, for it is often bilateral and may occur in connection with a similar attack on the lips and palate. Rarely an attack of true herpes may be found, usually on one side of the tongue, and this would not be expected to recur. The ordinary type is generally seen on the upper surface of the tip, and occasionally it may form a strip along the dossum. The writer is not inclined to allow that traumatic eruptions are true herpes, for he has only found them over the territory of the wounded nerve and not following a central course. In caries of the ribs and spine he thinks, on the other hand, that an affection of the posterior root ganglion occurs just as in true zoster. The same is true of symptomatic herpes in myelitis and tabes. Spontaneous zoster is, he holds, an acute specific disease of the nervous system analogous to anterior poliomyelitis, and due to an inflammation or hæmorrhage into the posterior root ganglion, followed by degeneration from the destruction of these ganglion cells.

[37] Med. Chron., December. [38] Boeck Practit., loc. cit. [39] Brit. Jour. Dermat., March, p. 95. [40] Lancet, March 23. [41] La Germanie Med., April 3. [42] Ibid., Dec. 26. [43] Allbutt's Syst., vol. viii., p. 623.

